# Supplementary material for: Using a standardized sound set to help characterize misophonia: The International Affective Digitized Sounds
Source: PLoS One. 2024 May 22;19(5):e0301105. doi: 10.1371/journal.pone.0301105 (PMC11111082; doi:10.1371/journal.pone.0301105)
Supplement: Attachment — Submitted filename: PONE-D-23-17567_REVIEW.docx [file pone.0301105.s001.docx]

This paper makes a compelling case for the need for standardized stimuli in misophonia research and offers a unique solution to that problem. The authors presented controls and misophonic individuals with sounds from IADS-2, a standardized set of emotional sound stimuli, and noted significant differences in how misophonic individuals viewed canonically positive and neutral sounds compared to controls. The authors rank the list of sounds by how large the difference in ratings is between misophonic individuals and controls, and subsequently propose the top 10 differentiating stimuli as “IADS-M” sounds for use in further research/therapy. This work contains some important points and interesting findings, but some restructuring and additional detail is necessary before it is suitable for publication.

Before listing specific comments, I want to disclaim my involvement in and draw the authors’ attention to the Free Open-Access Misophonia Stimuli (FOAMS) database, recently published in the Journal of Open Psychology Data, of which I am an author. This project similarly identifies the gap in misophonia literature that the authors discuss in the present manuscript – namely the lack of (and need for) a standardized set of misophonic stimuli – but attempts to bridge that gap in an orthogonal way. It is my opinion that the present manuscript still holds value and ought to be published. However, the authors should consider referencing FOAMS and/or differentiating their study from what FOAMS provides. I fully acknowledge that FOAMS may not have been available when the authors were preparing this manuscript (the FOAMS database was first posted online September 2022, and not published until August 2023), but the FOAMS paper may help emphasize the authors’ points. I am comfortable deferring to the handling editor’s judgment as to whether including FOAMS is appropriate.

Major comments:

1. Arguments in the manuscript would benefit from reframing:
   1.1. Use of self-report:
        Introduction paragraph 2 knocks the reliance on self-report measurements – for a variety of valid reasons – and advocates for more objective measures. However, the paradigm the authors employ is entirely self-report, and they discuss this as being a limitation in the Discussion. Thus, this argument in the introduction should be omitted/restructured.
        While asking participants to rate the sound of chewing is substantially better than asking them in text how they feel about chewing, it still suffers from response biases, etc. For example, the participants are asked to rate “arousal” on a scale from “calm” to “excited”, and I am interested in how much instruction/coaching was given to understand that question. (Anecdotally, I’ve struggled in my own work with getting participants to think of arousal non-sexually, and I personally don’t know if I’d rate even my most triggering sounds as “exciting”). Further, while the Similarity and Avoidance questions are interesting data points, I’m not convinced they accurately capture whether or not a misophonic person is triggered by that stimulus. (For instance, if I’m triggered by chewing sounds but the particular IADS-2 chewing stimulus doesn’t land for me, how would I answer “To what extent does this sound resemble the sounds in your everyday environment that most bother you?”)
        Perhaps, instead, the authors could comment on the wide array of self-reported prompts being asked in misophonia literature (e.g., discomfort, unpleasantness, tolerability, distress, annoyance, etc.) and highlight the utility of asking more standardized questions like valence/arousal, as doing so might help contextualize misophonia in relation to other auditory/psychiatric disorders that have data on valence/arousal.
   1.2. Positive/Neutral/Negative sounds:
        The use of these terms throughout the manuscript would benefit from some clarity/discussion. The key finding presented in the abstract is that misophonic participants rate pleasant and neutral sounds more negatively than controls do, which I think evokes a different image than what the study actually shows. Please correct me if I’m wrong, but it’s not so much that misophonic individuals hate classically pleasant sounds (which would be an interesting – but different – discussion), but that whoever provided normed ratings for IADS classified sounds as “positive” that are coincidentally also everyday sounds commonly reported as misophonic triggers. After staring at Table 2 for a while, my major takeaway is that what is positive vs. neutral vs. negative is super subjective (and probably differs by lived experience), and that there appears to be high heterogeneity within each category.
        Is the primary goal of this paper to compare ratings for sounds categorized as positive vs. neutral vs. negative, or to provide a list of sounds that differentiate misophonics from controls? If the former, I might suggest digging into the IADS-2 normed ratings a bit more and re-categorizing the sounds post hoc (e.g., highly positive, positive, positive-neutral, negative-neutral, negative, highly negative) so each category is a bit more homogenous. Alternatively, you could apply your own higher-level categories labels (e.g., “office”, “animal”, etc.), which I mention below (comment 3.2), and make a more specific content claim. However, if your main goal is to introduce the IADS-M, consider focusing less on the positive vs. neutral vs. negative differences and more on the stimuli as individual units.
        1.2.1. Sidenote: Perhaps these data also speak well to another issue I’ve struggled with articulating in misophonia research, which is “what sounds are truly neutral?!” These data demonstrate that sounds normed as Neutral (e.g., Brush Teeth, Clock) can also be bothersome to individuals with misophonia, so the authors might consider adding a word of caution against incorporating them as “control” sounds in future research…
   1.3. Utility:
        I’d suggest thinking more about who the audience of the IADS-M is. I discuss this in more detail below (comment 2.1.1), but the IADS-2 isn’t entirely open-access (especially in relation to something like FOAMS), so it might be less feasible for new researchers to incorporate these sounds into their research. Perhaps, instead, it’s a call to researchers or clinicians(?) who presently use the IADS-2 in auditory research to additionally screen for misophonia in their samples. Maybe the IADS-M can be a useful word of caution to researchers using IADS, that if you use those 10 sounds, you might inadvertently get unexpected results if your participants have misophonia. Regardless, Introduction and Discussion sections could be strengthened with a clearer focus of the utility of the IADS-M.
2. The manuscript is missing key information:
   2.1. IADS-2
        2.1.1. Accessibility: Throughout the manuscript, IADS-2 is referred to as “easily accessible.” The authors should include the steps required to obtain IADS-2, as not everyone is eligible to request them (and the process isn’t immediate), and consider caveating claims of accessibility. For instance, the IADS-2 request form requires that the “Requestor must be a PhD-holding faculty at a non-profit, degree-granting, academic institution,” with a “direct link to their official faculty profile on their institutional website that lists their credentials as a PhD-holding faculty”. Thus, I am unclear whether clinicians could actually utilize IADS-M sounds in their treatments, unless they have connection to a PhD-holding faculty member.
        2.1.2. Stimulus characteristics: The Method section should include more information regarding the content, length, volume, etc. of the IADS-2 sounds and the task paradigm (especially since Discussion paragraph 2 mentions “…the sounds in our study were standardized in terms of length, volume, duration, and other sound qualities”). Some questions I had include:
               2.1.2.1. Did sounds play automatically, or were they controlled by participants?
               2.1.2.2. Was the length of sound presentation sufficient to identify what the sound was? (cite previous misophonia research)
               2.1.2.3. Was there any time limit to submit ratings?
               2.1.2.4. What was the inter-stimulus interval?
               2.1.2.5. Were positive/neutral/negative sounds blocked or intermixed?
               2.1.2.6. How were the set of 56 sounds determined for each participant? (e.g., did they contain equal amounts of positive/neutral/negative stimuli?)
               2.1.2.7. How was volume standardized across participants? Did they wear headphones, or play the sounds aloud? Could participants have muted/changed volume midway through the experiment?
               2.1.2.8. Did participants know in advance that they would be hearing erotic/aversive stimuli? Could participants withdraw if they were uncomfortable?
        2.1.3. IADS-M: Unless I missed this, were the 10 IADS-M sounds ever mentioned/listed anywhere? Consider discussing them in text (e.g., Results, last paragraph) and/or denoting them in Table 2. Further, why 10?
   2.2. Participants:
        2.2.1. More detail on group inclusion criteria would be useful. I’m less familiar with the MQ, but where do the cutoffs of 2 on the Symptom and Emotions/Behavior Scales come from? It initially reads like a very low cutoff – perhaps include the max value that the scales are out of for context.
        2.2.2. Why was the control group recruited before the misophonic group? Do the samples differ unintentionally due to the passage of time?
        2.2.3. If inclusion criteria narrowed after 225 controls were recruited, why did the final control group include 245 participants? What determined the 225 (initial) stopping number – was there an a priori power analysis?
        2.2.4. Why were there unequal numbers of participants in the control vs. misophonia groups? While equal gender splits across the entire sample is impressive, looking at Table 1, it does not hold true within sample; did the groups differ significantly in terms of gender, age, etc.?
        2.2.5. Starting with 2550 participants and ending with 377 seems like a huge drop off. Can you comment on why? What proportion of the excluded participants failed the screening vs. attention checks?
        2.2.6. How much were participants compensated to participate?
        2.2.7. Ethics: I trust that if the study procedures were approved by the university IRB then this is fine, but the current phrasing of the ethics statements (e.g., “implied consent”) read extremely sketchy. Consent should be obvious, not implied. As above (comment 2.1.2.8), were participants informed about the potentially aversive nature of the study? Could they withdraw without penalty?
3. The manuscript would benefit from additional analyses/visuals:
   3.1. Additional analyses would be helpful to support the idea that IADS-M sounds are useful for differentiating individuals with misophonia. For instance, does a classifier built on responses to these sounds significantly predict group membership? Do the ratings of valence/arousal/etc. of these sounds in particular correlate the strongest with MQ score?
   3.2. Consider additional category breakdowns (e.g., “office” sounds, as referenced in Discussion), instead of just positive/negative/neutral. Are the results that individuals with misophonia respond with lower valence to normed positive stimuli driven by any category in particular? Doing so might illuminate a more nuanced understanding of misophonic triggers.
   3.3. Consider adding summary figures to visually parse the results, as otherwise the Results section text is dense. Additionally, unless you have specific predictions about differences between sound types collapsed across group membership, consider omitting these paragraphs (i.e., Results paragraphs 5, 8, etc.) from the Results section since they aren’t really discussed further. Perhaps they could be moved to a table or Supplement.
   3.4. Clarify statistics/multiple comparison corrections:
        3.4.1. I am happy to see the authors were conscious of corrections and included a Bonferroni-corrected alpha in Results paragraph 4. However, it is unclear how often/which results were corrected (e.g., is Results paragraph 7 corrected? Paragraph 10?), since the post hoc stats are still reported at an alpha of 0.05.
        3.4.2. Table 4: Are these correlations corrected for multiple comparisons?
        3.4.3. Results, paragraph 1: Were correlations run for each subscale of the MQ, or only Total scores? If the former, consider adding the individual subscale correlations to Table 4, so results reported in text can be visualized in context of other variables.

Minor comments:

4. Needs more citations:
   4.1. Introduction, paragraph 8: Throughout
   4.2. Method, Measures and Materials: Provide example studies for “commonly used in research of emotion and attention”
   4.3. Discussion, paragraph 2: Provide examples of “Like previous studies,”
   4.4. Discussion, paragraph 3: Provide examples of “extend previous research” and “Unlike previous studies”
   4.5. Discussion, paragraph 4: Throughout. Link your findings of environmental/office sounds (or non-oral/nasal sounds in general) being bothersome with previous literature that reports similar results.
   4.6. Discussion, paragraph 5: Throughout. Also, for what it’s worth, Hansen et al. (2021) does use bird sounds as a stimulus, although it wasn’t found to be tremendously bothersome (to either individuals with misophonia or controls).
   4.7. Throughout: consider referencing FOAMS
5. Line edits:
   5.1. Abstract: Add a comma after “(IADS-2)”, or remove the comma after “Bradley and Lang”
   5.2. Introduction, paragraph 1: I’d recommend adding “in 2013” somewhere in the last sentence to contextualize Schroder et al.’s work and clarify the timeline of misophonia research
   5.3. Introduction, paragraph 3: Start a new paragraph at “In contrast, if a more standardized approach…”
   5.4. Introduction, paragraph 5: I’d recommend reframing this paragraph, as “most studies have been limited to study team expertise and a review of the literature” feels like an attack on otherwise good practices. I think your underlying point is valid and important – that if we don’t branch out and try other sounds, we may be missing out on other crucial trigger sounds that aren’t as commonly reported – but it could be made in a different way. For example, I’ve gone for something like “participants report a wide variety of trigger sounds, but only a small subset of sounds is actually used in studies”. Also, I’d recommend removing “the tail wagging the proverbial dog”, as it feels a little too colloquial.
   5.5. Introduction, last paragraph: Rephrase “estimated avoidance of sounds avoid them”
   5.6. Current Study, last paragraph: Add “people” to “…avoidance of these sounds than those [people] without misophonia” (I initially read it as “…these sounds than those sounds…”)
   5.7. Method, Participants: Change “data was” to “data were”
   5.8. Method, Data Integrity Check: Consider renaming “attention check” (“technology check”?), since the two questions listed don’t explicitly measure participants’ ability to direct or maintain attention.
   5.9. Method, Measures and Materials: As with MQ, introduce acronyms in bold following the name of the measure at the start of each paragraph. Then, just refer to them all with acronyms in the last sentence of Method, Procedure.
   5.10. Method, Measures and Materials, Positive and Negative Affect Scale: Rephrase the sentence “Cronbach’s alpha for the Positive Affect scale is reportedly 0.86-0.9 and 0.84-0.87 for the Negative Affect Scale” with parallel structure
   5.11. Method, Procedure: Add “as” to “…as well [as] self-report measures”
   5.12. Method, Procedure: Give anchors/more detail to “a Likert scale ranging from 1-9”. (e.g., is 1 low or high arousal/valence?)
   5.13. Method, Data Analytic Plan: Swap the order of the first 2 paragraphs (otherwise “these analyses” and “all four ANCOVAS” are confusing without explanation)
   5.14. Method, Data Analytic Plan (and elsewhere): Replace “Table X”
   5.15. Results, paragraph 1: “the MQ Symptom Subscale was significantly negatively correlated with the mean negative valence, arousal, similarity, and avoidance scores (*rs* = -.34 .34, .21, and .35,…” – was MQ negatively correlated with all four measures, or just the first? Rephrase the text or the r-values accordingly.
   5.16. Results, paragraph 8: Remind the reader why p = 0.024 is not significant (i.e., that corrected alpha is 0.017)
   5.17. Results: For stats in which both mean ratings and CI are reported in parentheses, keep consistent where parentheses are reported -- e.g., (.205 (95% CI, -.00 to .41; *p* > .05) is missing a parenthesis
   5.18. Results: Replace all instances of “p = 0.000” with “p < 0.001”
   5.19. Results: Keep reporting of p-values consistent. E.g., sometimes exact values are reported (e.g., p=.024, p=.173), sometimes approximation (e.g., p<.05, p>0.05)
   5.20. Discussion, paragraph 5: Rephrase “These sounds were not obviously triggering, as they were not oral-facial…” At face value, I think it’s been well-established that non-orofacial sounds can also be triggering; if this was your point, I’d remove this sentence. However, if your point is that this type of sound wouldn’t classically be used as a “trigger” stimulus because it’s not orofacial and most literature still prioritizes orofacial sounds, I think that’s an important discussion point and should be rephrased for clarity.
   5.21. Discussion, last paragraph: Since your research question doesn’t involve development or measurements over time, cross-sectional vs. longitudinal design limitations do not feel necessary. I’d recommend removing the first bit and starting the paragraph at “Finally, the diversity of participants…”
   5.22. Table 1: Report a consistent number of decimal spots for all data values. Also, is the percentage for White individuals with Misophonia cut off?
   5.23. Table 2: Why are there two columns for Valence?
   5.24. Table 4: Keep the anchor words for the ratings consistent throughout the manuscript (e.g., the text says “calm/excited” but this caption says “relaxing or exciting”).
   5.25. Table 4: Change “7. Ave Similarity…” to “7. Avg Similarity”. Also, the caption to Table 4 would be easier to parse with the sentences beginning “Avg Ratings for…” numbered as in the table and split on separate lines.

Overall, thanks for the great work! I look forward to seeing an updated version.

Signed,

Heather Hansen
