# Supplementary material for: Using a standardized sound set to help characterize misophonia: The International Affective Digitized Sounds
Source: PLoS One. 2024 May 22;19(5):e0301105. doi: 10.1371/journal.pone.0301105 (PMC11111082; doi:10.1371/journal.pone.0301105)
Supplement: Attachment — Submitted filename: Response to Reviewers.docx [file pone.0301105.s003.docx]

**RESPONSE TO REVIEWERS**

Response: We appreciate the editors’ and reviewers’ time and consideration of our manuscript. We believe our responses below sufficiently address the concerns and feedback shared. Overall, we believe the changes made have significantly strengthened the manuscript and appreciate your consideration of the revised manuscript for publication in PlosOne.

Reviewer #1: The authors did a great job responding to reviewer comments. The manuscript is much clearer now, especially throughout the results section. However, in my view the tables are still unnecessarily confusing. Table 2 still has the column heading "valence" even though what's shown in that column are z-scores representing group difference in valence ratings. Perhaps a better way to label that column would be something like "group difference (z-scores)" or something like that? The description in the manuscript text (p. 8) states "higher rankings (e.g., 1, 2) reflect the sounds that most differentiate individuals with misophonia from controls" but this is confusing because there is no actual ranking provided in the table (like 1 or 2) and the numbers in the "valence" column range from ~-4 to 1. Now that the authors removed the language about the IADS-M as the top 10 differentiating sounds, it's also unclear which sounds "count" as part of the IADS-M. Is it all of the sounds in Table 2? Is there a threshold you would recommend for other researchers that distinguishes IADS-M from the rest of the IADS sounds? Tables 3 and 4 are not terribly helpful with the stimuli listed by number and without any description, plus they include information redundant with Table 2. In my view it would be far better to provide an additional two columns for Table 2-- one for each group's mean valence ratings for each stimulus. I don't see why the tables need to include the other dependent measures given that valence was the main variable that differentiated the two groups. If these tables could be made just a little clearer, I do think this paper would be improved and would make a very nice contribution to the literature.

Response 1: Thank you for pointing out that we were not clear enough in labeling and explaining the tables! Valence scores for both groups are now in Table 2, and the column heading has been corrected. Tables 3 and 4 have been removed. The IADS-M simply refers to the reordered list, with no particular threshold recommendation- this would be up to researchers to decide how many sounds they would like to use for their purposes.

Editors Comment 1: As the authors are finalizing their manuscript, I see one minor piece for the authors to update, which is making sure the citations added in the revision (specifically Orloff et al., 2023; Frank et al., 2020; Simner et al., 2021) are incorporated into the reference list.

Response 1: Thank you for pointing out this mistake! The reference list has been amended.
